# IMPORTANT IMPROVEMENTS TOWARD EQUITY IN PROSTATE CANCER SURVIVAL BETWEEN WHITE AND BLACK MEN

**DOI:** 10.1093/geroni/igad104.3424

**Published:** 2023-12-21

**Authors:** Ray Merrill, Ian Gibbons

**Affiliations:** Brigham Young University, Provo, Utah, United States; Brigham Young University, Provo, Utah, United States

## Abstract

Prostate cancer survival is significantly influenced by tumor stage, age, and marital status at diagnosis. There is also evidence that cases diagnosed prior to age 40 and after age 80 years have a poorer prognosis. While Blacks have historically had lower prostate cancer survival than whites, it is not clear whether this difference still exists and if it varies across the levels of tumor stage, age, or marital status at diagnosis. These issues are addressed in this study. Analyses are based on Surveillance, Epidemiology, and End Results (SEER) data to obtain 5-year relative survival (5-YRS) estimates. Cases reflect 2000-2015 and follow-up extends through 2020. Over the study period 5-YRS decreased for Whites and increased for blacks, such that by 2015 the 5-YRS was about the same between races. Multiple regression models with race, age, and marital status simultaneously estimated showed that 5-YRS in Whites versus Blacks was not significantly different for local/regional stage or distant stage. 5-YRS in married (or living with a partner) was 3.02 higher for local/regional stage but not significant for distant stage. 5-YRS was significantly lower in the age groups < 40 and ≥80 for local/regional stage but were highest in the age group < 40 and significantly fell with older age for distant stage. Race did not modify the relationship between 5-YRS and tumor stage, age, or marital status at diagnosis. Thus, while tumor stage, age, and marital status at diagnosis are important predictors of 5-YRS for prostate cancer, White and Black inequality no longer exists.

